# Exploring the structure of the shot effectiveness model for elite table tennis players

**DOI:** 10.1186/s13102-023-00736-x

**Published:** 2023-10-06

**Authors:** Qing Yang, Mu-zi Li, Zheng Zhou, Hui Zhang

**Affiliations:** 1 School of Physical Education, Soochow University, 50 Donghuan Road, Suzhou, 215021 Jiangsu Province China; 2 Department of Sport Science, College of Education, Zhejiang University, 866 Yuhangtang Road, Hangzhou, 310058 Zhejiang Province China

**Keywords:** Table tennis, Shot effectiveness, Model structure, Multiple regression, Racket sports

## Abstract

**Background:**

Currently, technical and tactical analysis has become an indispensable task for sport in many countries. Many studies analysed players’ specific technical and tactical factors, but it is rare to quantitatively analyse the importance of table tennis players’ shot effectiveness. This is the first study to propose the new concept of “shot effectiveness model”, and the purpose of this study is to explore the structure of the shot effectiveness model for elite table tennis, including the importance degree of shot effectiveness, and the relationship between them.

**Methods:**

258 matches were selected between the top 50 players in the world from 2019 to 2021 as samples. Multiple regression analysis was used to obtain the standard regression coefficients and game simulation, and the total decision coefficient (TDC) was used to evaluate the importance degrees of shot effectiveness (SE) on match results.

**Results:**

(1) There was little difference in the importance degree of each shot effectiveness between men and women players. (2) The importance degree of the first and third shots (SE_1_), the second and fourth shots (SE_2_), the fifth and after shots (SE_3_), and the sixth and after shots (SE_4_) for both men and women players account for approximately 25%, 35%, 22%, and 16% respectively. (3) There was little difference in the importance degree of each shot effectiveness between Chinese women players and women players from other countries and regions with the same importance order of SE_2_ > SE_1_ > SE_3_ > SE_4_. However, the structure of the shot effectiveness model for men players was quite different from that for women players. (4) There is a compensation effect between shot effectiveness of table tennis players, and the total evaluation score of 12 and 13 is the dividing line for success or failure in both men and women matches.

**Conclusions:**

TDC could well reflect the important degrees of each shot effectiveness in various ways on winning probability in table tennis matches. And this study compared the importance of several types of players’ performance on the probability of winning a match. In addition, we found that there is a compensation effect between shot effectiveness of table tennis players, and the magnitude of this effect will vary according to the type and level of shot effectiveness.

## Background

Tian [1] stated that the competitive ability of players in sports is determined by five aspects: techniques, tactics, physical fitness, psychology, and intelligence. Hughes and Bartlett [2] classified the performance indicators of different sports into three categories: technical, tactical, and biomechanical. Due to the complexity of techniques and the flexibility of tactics in table tennis, techniques and tactics are the leading factors for table tennis players. Therefore, research on players’ techniques and tactics has always been the core work of table tennis in China. Currently, technical and tactical analysis has also become an indispensable task for sport in many countries with good performance, such as China, Japan, and Germany, in preparing for international tournaments [3, 4].

Knowledge of the impact of table tennis’s various technical and tactical elements on performance is crucial for training and competition [3, 5–7]. For nearly 20 years, some advanced analysis models have been applied in this field. For example, the artificial neural network model [8–10], association rules model [11, 12], expert knowledge model [13, 14], and Markov chain model [15–17] were used to explore the impact of technical-tactical/tactical elements. Some researchers have proposed new methods to improve the accuracy and rationality of analysis indicators [7, 18–20] and, on this basis, have compared the performance of players in Asian / China and other countries and regions to indirectly reflect the importance of various technical and tactical factors [21, 22]. These studies have explored different perspectives on table tennis techniques and tactics through one or more indicators (usage rate, scoring rate, effectiveness), but most of them were based on specific technical or tactical behaviour that does not reflect the overall essential characteristics of the winning rules of table tennis.

Among many methods, the “three-phase evaluation method” is the most classical and widely used in Chinese national team preparation for world competitions [23]. On this basis, Yang and Zhang [24] proposed the “four-phase evaluation method”, which has made some improvements to the “three-phase evaluation method” and now is widely used in practice [25–28] and as reference in tennis and badminton events [29, 30]. In addition to being simple and easy to operate, an important reason this method has been widely adopted by professionals in practice is that these “phased evaluation methods” capture the core of table tennis matches by combining two or more shots.

Therefore, how important is shot effectiveness for the success of a game? Practical experience of table tennis training has stressed the importance of the first three shots for many years, how important of these in the end? Is there a difference in the importance of shot effectiveness between men and women players? Compared with players from other countries or regions, is the shot effectiveness of Chinese players unique? There are many studies citing relevant theoretical methods to analyse players’ specific technical and tactical factors [7, 22, 26], but it is rare to quantitatively analyse the importance degree of table tennis players’ shot effectiveness and its relationships. On this basis, this study propose the new concept of “shot effectiveness model” and that is also an innovation point of this study. The purpose of this study is to explore the structure of the shot effectiveness model for elite table tennis, and the following hypotheses are posited: (a) there exists a distinction in the structure of shot effectiveness between men and women players; (b) noticeable differences emerge in the structure of shot effectiveness between Chinese players and those from other countries and regions.

## Methods

### Samples

For this study, a total of 258 matches were selected as samples, encompassing the top 50 players globally from 2019 to 2021. This selection comprised 124 matches for men players and 134 matches for women players, all of whom were devoid of players employing a chopping style. Among these, there were 64 and 67 matches featuring 9 men and 10 women Chinese players, respectively, while 60 and 67 matches included 26 men and 16 women players from other countries such as Japan, Korea, Germany, Brazil, Sweden, England, India, Romania, Singapore, and regions like Chinese Hong Kong and Chinese Taipei. The matches encompassed events such as the World Cup, World Championships, Asian Cup, and the Olympic Games, among others (Table 1).

All match videos were sourced from television broadcasts or the ITTF website (https://www.ittf.com/rankings/) and WTT website (https://worldtabletennis.com/ rankings). The study received approval from the local institutional ethics committee.

**Table 1:** The information of the 258 matches

Type of tournaments	Men (N)	Women (N)	Best of 5 or 7 games	Men (N)	Women (N)	Level of draws	Men (N)	Women (N)
World Championship	6	5	Best of 5 games	11	9	Finals	9	19
World Cup	20	14	Best of 7 games	113	125	Semi finals	21	18
Olympic Games	4	4				1/4 finals	31	32
Asian Championship	6	4				1/8 finals	41	50
World Tour Open	84	84				1/16 finals	6	1
World Tour Grand Finals	2	20				Bronze finals	2	0
Asia Pacific Table Tennis League	2	0				First Round	10	12
WTT Champions	0	1				Second Round	4	2
Asian Cup	0	2						

### Research design

#### Structure of the shot effectiveness model in table tennis

**Fig. 1:**
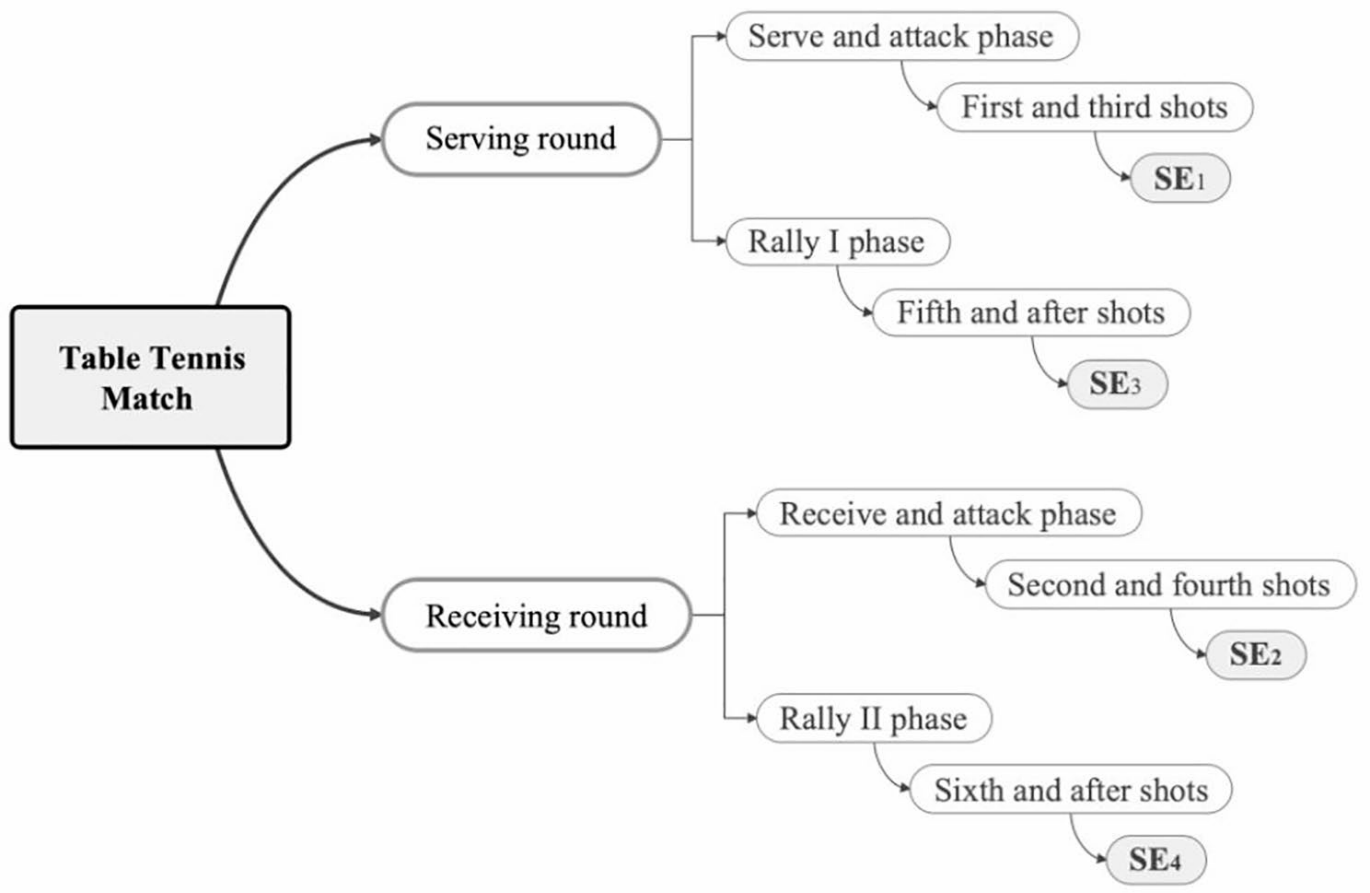
Structure of the shot effectiveness model of table tennis

Following the related literature [24], the shot effectiveness (SE) model of tablet tennis matches can include four indicators: the effectiveness of the first and third shots (SE_1_), the effectiveness of the second and fourth shots (SE_2_), the effectiveness of the fifth and after shots in the serving round (SE_3_), and the effectiveness of the sixth and after shots in the receiving round (SE_4_). Figure 1 shows the shot effectiveness model of table tennis.

#### Computation of shot effectiveness

There are three common methods to calculate the shot effectiveness of table tennis. Zhang, Liu [19] and Zhou [31] calculated shot effectiveness through the relationship between the scoring rate and usage rate, while Tamaki et al. [7] computed shot effectiveness by subtracting the scoring rate and loss rate. Because the shot effectiveness proposed by Tamaki et al. was more concise and easy to understand, his calculation method was used in this study. According to Table 2, the calculation formulas of different shot effectiveness and the winning probability (WP) were as follows:

**Table 2:** Shot, scoring, losing and their codes in table tennis

Round	Shot	Scoring code	Losing code	Neutral code
Serving	1	A^+^	A^−^	A
	3	B^+^	B^−^	B
	Fifth and after shots	C^+^	C^−^	C
Receiving	2	D^+^	D^−^	D
	4	E^+^	E^−^	E
	Sixth and after shots	F^+^	F^−^	F

Note: Neutral means that the shot is neither scored nor lost

1 $${SE}_{1}=\frac{({A}^{+}+{B}^{+})-({A}^{-}+{B}^{-})}{{A}^{+}+{A}^{-}+\text{A}+{B}^{+}+{B}^{-}+\text{B}}$$

2 $${SE}_{2}=\frac{({D}^{+}+{E}^{+})-({D}^{-}+{E}^{-})}{{D}^{+}+{D}^{-}+\text{D}+{E}^{+}+{E}^{-}+\text{E}}$$

3 $${SE}_{3}=\frac{ {C}^{+}-{C}^{-}}{{C}^{+}+{C}^{-}+C}$$

4 $${SE}_{4}=\frac{{F}^{+}-{F}^{-}}{{F}^{+}+{F}^{-}+F}$$

5 $$WP=\frac{{A}^{+}+{B}^{+}{C}^{+}{D}^{+}+{E}^{+}+{F}^{+}}{{A}^{+}+{A}^{-}+{B}^{+}+{B}^{-}+{C}^{+}+{C}^{-}+{D}^{+}+{D}^{-}+{E}^{+}+{E}^{-}+{F}^{+}+{F}^{-}}$$

#### Multiple regression analysis

Multiple regression analysis is used to obtain the standard regression coefficients and game simulation. Taking *SE*_*1*_, *SE*_*2*_, *SE*_*3*_, and *SE*_*4*_ as independent variables and *WP* as the dependent variable, two regression models for men and women players were established, as shown in Formula 6.

6 $$WP = {b_0} + {b_1}S{E_1} + {b_2}S{E_2} + {b_3}S{E_3} + {b_4}S{E_4} + e$$

In this equation, “*b*_*0*_” is a constant, “*b*_*1*_, *b*_*2*_, *b*_*3*_, *b*_*4*_” are pending parameters, and *e* is the error term.

#### Computation method of the total decision coefficient

To our knowledge, the total decision coefficient (TDC) was initially employed in this study to investigate the structure of the shot effectiveness model for players. TDC is the product of the correlation coefficient of the independent variable and the dependent variable and the standard regression coefficient. TDC shows the percentage of variation in the dependent variable that can be explained by the independent variable, that is the total decision degree of each independent variable through various ways on the dependent variable [32]. From this, the importance degree of various shot effectiveness to win the match can be obtained through the equation as follows:

7 $${TDC}_{i}={SRC}_{i}\times {\text{R}}_{iwp}\times 100\text{\%}$$

In Eq. 7, *i* is denoted as the number of shot effectiveness, *i* = 1, 2, 3, 4. *SRC*_*i*_ represents the standard regression coefficient of each independent variable in a multiple regression model. *R*_*iwp*_ represents the correlation coefficient between an independent variable (*SE*_*i*_) and the dependent variable (*WP*).

### Data collection and modelling

A table tennis data collection and analysis system was developed and has been utilized in related research, demonstrating favorable objectivity [18]. Two experienced table tennis players acted as data collectors and employed this system to semi-automatically gather all the necessary data. The correlation analysis and the multiple regression models were performed using SPSS version 24.0 software (SPSS Inc., Chicago, IL, USA) for Windows.

Five matches were randomly selected from the above samples, and another collector observed and recorded them. The result of Cohen’s kappa statistics (Inter-Rater-Agreement) [33] showed that the Cohen’s kappa values (k) of the observation indicators were equal to 1, which indicates that the objectivity of the observation indices was confirmed.

## Results

### Basic data

Table 3 shows the mean and standard deviation of the indices of table tennis players in the two models. Pearson correlation coefficient interval between all independent variables is [0.141, 0.374], which shows that these indices have low or no correlation.

**Table 3:** Mean and standard deviation of the indices in two models for elite players

	Men (*n* = 124)	Women (*n* = 134)
SE_1_	0.077 ± 0.073	0.070 ± 0.079
SE_2_	0.008 ± 0.093	0.006 ± 0.107
SE_3_	-0.075 ± 0.135	-0.069 ± 0.159
SE_4_	-0.135 ± 0.150	-0.114 ± 0.175
WP	0.503 ± 0.078	0.501 ± 0.100

### Multiple regression model and TDC

Table 4 shows that the Durbin-Watson test values of the two regression models were all close to 2.0, and their residuals were independent of each other. Assessment of multicollinearity revealed that the VIF was between 1.0 and 2.0 for all independent variables in all models, indicating the absence of multicollinearity. Assessment of normality revealed that the residuals basically conformed to a normal distribution. The two regression models were statistically significant (*P* < 0.001), and the adjusted *R*^*2*^ values were 0.977 and 0.978 respectively. According to the above results, the multiple regression equation of men and women players can be written as follows:

8 $${Y_m} = 0.502 + 0.440{X_1} + 0.411{X_2} + 0.210{X_3} + 0.149{X_4}$$

9 $${Y_f} = 0.495 + 0.477{X_1} + 0.421{X_2} + 0.194{X_3} + 0.144{X_4}$$

**Fig. 2:**
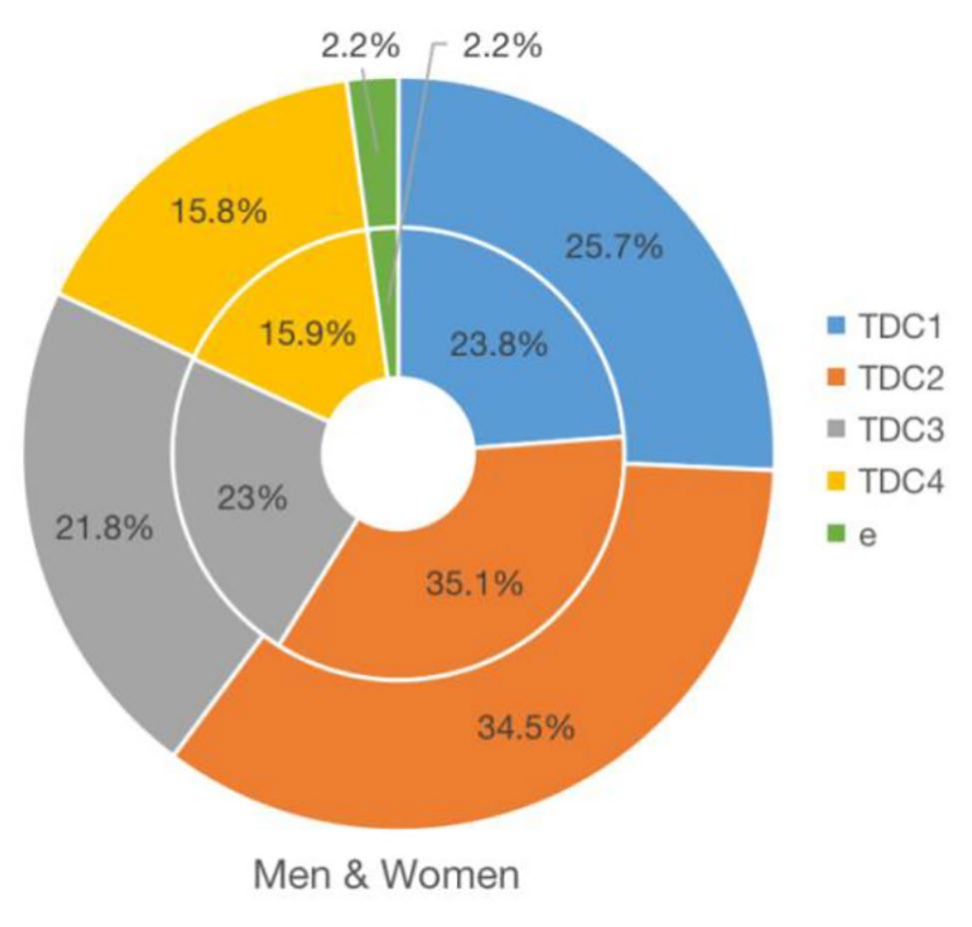
TDC values of the shot effectiveness for men and women players Note: The outer ring shows the TDC values of women players, and the inner ring shows the TDC values of men players

According to Eq. 7, the TDC of each shot effectiveness for men and women players can be obtained in Fig. 2. The results show that TDC values for both men and women players are similar, as TDC_1_ for men and women players are 23.8%, 25.7%; TDC_2_ for men and women players are 35.1%, 34.5%; TDC_3_ for men and women players are 23%, 21.8%; TDC_4_ for men and women players are 15.9%, 15.8%, respectively. TDC values for both men and women players are all ranked in the following order: TDC_2_ > TDC_1_ > TDC_3_ > TDC_4_.

**Table 4:** Results of regression models for elite players

	B	β	*t*	*p*	95%CI	*Adjusted R* ^*2*^	*F*	*F(sig)*	Durbin-Watson
Men						0.977	1287.006	0.000	1.651
Constants	0.502		243.535	0.000	0.498, 0.506				
SE1	0.440	0.409	28.933	0.000	0.410, 0.470				
SE2	0.411	0.488	33.242	0.000	0.387, 0.436				
SE3	0.210	0.362	24.554	0.000	0.193, 0.226				
SE4	0.149	0.286	19.657	0.000	0.134, 0.164				
Women						0.978	1410.732	0.000	1.745
Constants	0.495		217.067	0.000	0.490, 0.499				
SE1	0.477	0.377	26.015	0.000	0.440, 0.513				
SE2	0.421	0.454	30.733	0.000	0.394, 0.448				
SE3	0.194	0.310	20.444	0.000	0.176, 0.213				
SE4	0.144	0.254	17.345	0.000	0.128, 0.161				

To further explore the structural differences between different players’ shot effectiveness models, four regression models for Chinese men and women players, and men and women players from other countries or regions were established according to Formula 6. The results showed that the residuals of the four models were independent of each other, and basically conformed to a normal distribution. All independent variables had no multicollinearity with the VIF between 1.0 and 2.0. The four regression models all have significant significance (P < 0.001), the adjusted R^2^ are all above 0.950, and the independent variables in these models all have significant significance (P < 0.001). According to Eq. 7, the TDC of each shot effectiveness for these four types of players can be obtained in Fig. 2.

**Fig. 3:**
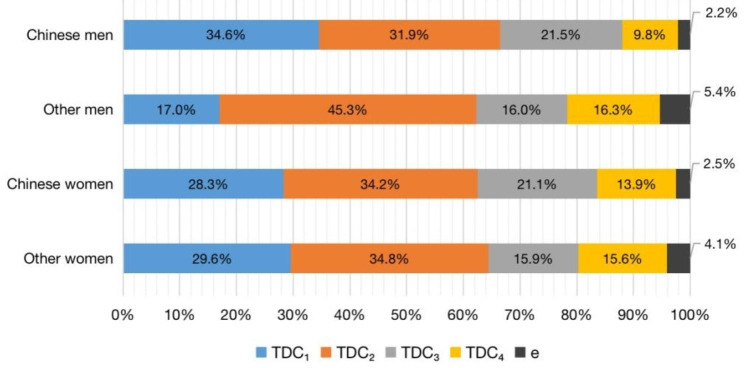
TDC values of the shot effectiveness for four types players

The results show that TDC values for Chinese women players and women players from other countries and regions are similar, with the same importance order of TDC_2_ > TDC_1_ > TDC_3_ > TDC_4_. In contrast, men players display differences in the significance of shot effectiveness. TDC_1_ for Chinese men players and men players from other countries and regions are 34.6% and 17%, respectively; TDC_2_ for Chinese men players and men players from other countries and regions are 31.9% and 45.3%, respectively; TDC_3_ for Chinese men players and men players from other countries and regions are 21.5% and 16%, respectively; and TDC_4_ for Chinese men players and men players from other countries and regions are 9.8% and 16.3%, respectively.

### Game simulation analysis of different combinations of shot effectiveness levels

The established multiple regression (Eqs. 8, 9) is used to simulate the games of the combination of different shot effectiveness levels. Taking 80%, 50%, and 20% as the percentile split points, each shot effectiveness can be divided into four levels. The evaluation criteria for men and women players are shown in Table 5.

**Table 5:** Evaluation criteria for shot effectiveness of elite player

	Excellent	Good	General	Poor	Minimum
Men	SE_1_ ≥ 0.143	0.143 > SE_1_ ≥ 0.071	0.071 > SE_1_ ≥ 0.018	SE_1_ < 0.018	-0.103
	SE_2_ ≥ 0.079	0.079 > SE_2_ ≥ 0.010	0.010 > SE_2_ ≥ -0.070	SE_2_ < -0.070	-0.236
	SE_3_ ≥ 0.027	0.027 > SE_3_ ≥ -0.073	-0.073 > SE_3_ ≥ -0.189	SE_3_ < -0.189	-0.441
	SE_4_ ≥ 0.000	0.000 > SE_4_ ≥ -0.144	-0.144 > SE_4_ ≥ -0.256	SE_4_ < -0.256	-0.700
Women	SE_1_ ≥ 0.130	0.130 > SE_1_ ≥ 0.065	0.065 > SE_1_ ≥ 0.000	SE_1_ < 0.000	-0.143
	SE_2_ ≥ 0.094	0.094 > SE_2_ ≥ 0.000	0.000 > SE_2_ ≥ -0.076	SE_2_ < -0.076	-0.254
	SE_3_ ≥ 0.080	0.080 > SE_3_ ≥ -0.077	-0.077 > SE_3_ ≥ -0.212	SE_3_ < -0.212	-0.462
	SE_4_ ≥ 0.046	0.046 > SE_4_ ≥ -0.108	-0.108 > SE_4_ ≥ -0.265	SE_4_ < -0.265	-0.538

**Fig. 4:**
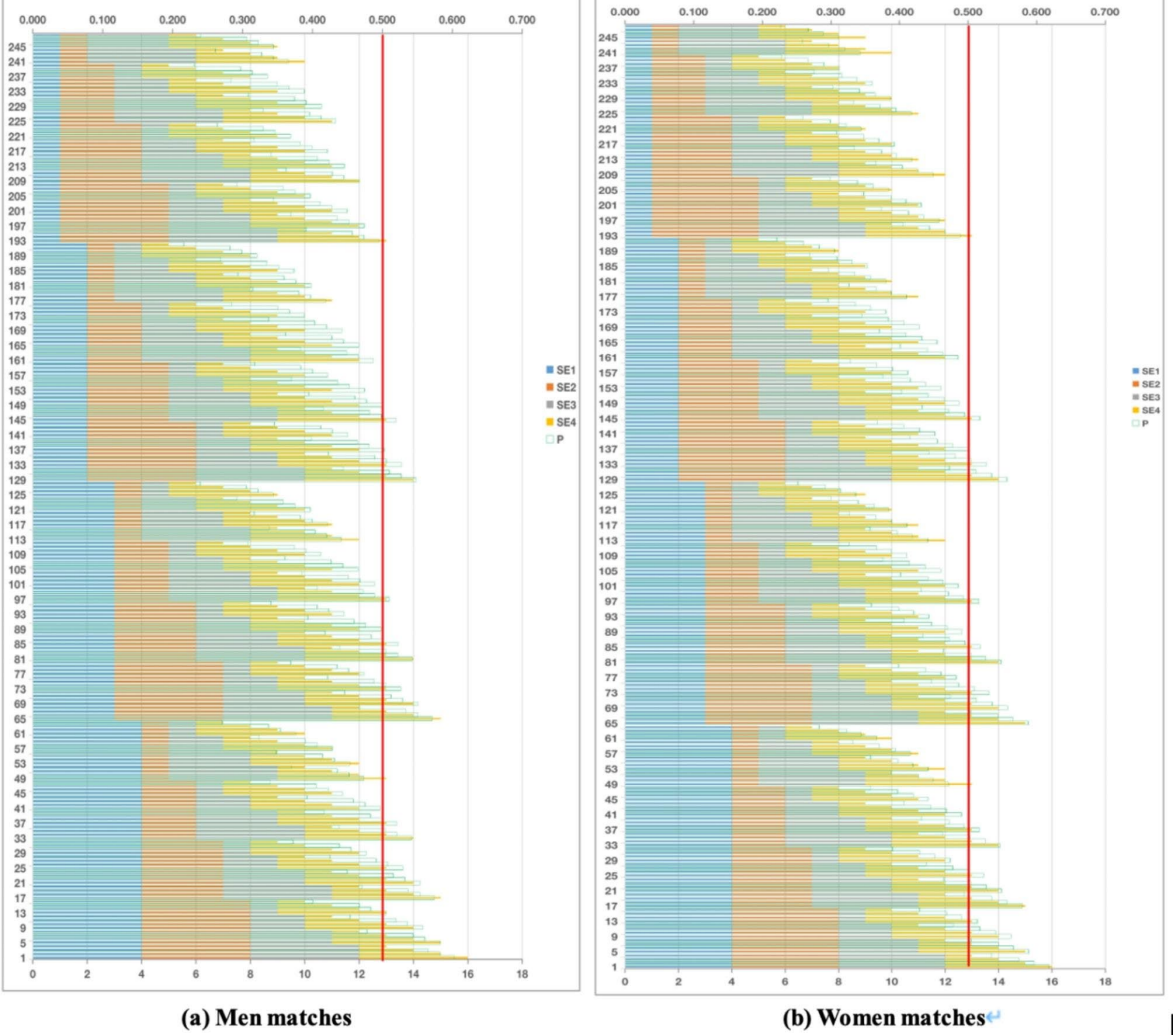
Simulation of 256 combination modes in men and women matches Note: P: winning probability of simulated game with different shot effectiveness combinations; x-axis represents the evaluation score; x secondary axis represents the winning probability; y-axis represent the 256 combination modes

There are 256 combinations with four shot effectiveness, each with four levels. The lower limit value of each evaluation criterion (the minimum value of each group indicator as the lower limit value in the “poor” level, seen in Table 5) was brought into the established multiple regression equation as an independent variable. The output results of multiple regressions are the lowest winning probability of 256 combined games with different shot effectiveness levels. Let us assign 4, 3, 2, and 1 points to the evaluation criteria of “excellent”, “good”, “general”, and “poor” respectively. Then the highest total evaluation score is 16 with the combination “excellent-excellent-excellent-excellent”, and the lowest is 4 with the combination “poor-poor-poor-poor”, as shown in Fig. 4.

Taking 0.5 as the standard line, when the winning probability of the combination mode is greater than or equal to 0.5, it is regarded as winning, while below 0.5 is regarded as a failure. The total evaluation score of 12 and 13 is the dividing line for success or failure in men matches. The game will win when the total evaluation score is higher than 13, while the game will lose when it is lower than 12. When the evaluation total score is equal to 13, 85% of the combination mode could get winning, and the “3 excellent 1 poor” combination mode is the lowest probability mode for winning. Except for the “excellent-excellent-poor-excellent” combination, other combinations of “3 excellent 1 poor” would all fail. When the total evaluation score is equal to 12, 35% of the combinations could win. The combination mode of “2 excellent 1 good 1 poor” is the lowest probability mode for winning, in which all the combinations with “poor” would all fail.

The total evaluation score of 12 and 13 is also the dividing line for success or failure in women matches which is the same as the men matches. When the total evaluation score is equal to 13, 90% of the combination mode could get winning. The combination modes of “excellent-poor-excellent-excellent” and “poor-excellent-excellent-excellent” would fail, and other combination modes would win. When the total evaluation score is equal to 12, 39% of the combination could get winning. The combination mode of “2 excellent 1 good 1 poor” still has the lowest probability of winning. Except for the combination modes of “good-excellent-excellent-poor” and “excellent-excellent-good-poor”, all other combination modes of “2 excellent 1 good 1 poor” will be lost.

## Discussion

### Structural differences between different players’ shot effectiveness models

#### Gender difference

The results of TDC values (Fig. 2) imply that there was little difference in the importance degree of each shot effectiveness between men and women players. However, SE_1_, SE_2_, SE_3_, and SE_4_ differ in their respective degrees of importance. SE_1_ and SE_2_ have advantages in the game sequence, and those who grasp the opportunity and initiative early in the first four shots will have a higher winning probability. SE_2_ was more important than SE_1_, the possible reasons are related to technical innovations in receiving (Twist, a backhand attack technique used primarily in receiving) and changes in competition rules of the International Table Tennis Federation. Since the implementation of the plastic ball in 2014, the velocity and spinning of the player’s serving ball have decreased so that opponents can be more likely to attack when receiving the ball [34].

SE_3_ and SE _4_ are not as important as SE_1_ and SE _2_ for table tennis players. The sum of the importance of SE_1_ and SE_2_ for both men and women players accounts for approximately 60%, and that of SE _3_ and SE _4_ accounts for approximately 40%. The importance of SE_3_ for both men and women players was higher than that of SE_4_. The biggest difference between them is that SE_3_ more easily adopts the active rally, while SE_4_ has a more passive rally, which may mean that at a current technical and tactical level, the active attack still plays a major role in the rally phase, that is, the rally should be “fierce” and “attack”.

These results basically support the traditional experience knowledge of table tennis [35–37], even after the rule reformed by International Table Tennis Federation (ITTF), there were also relevant studies to support this result [7, 38, 39]. However, Wenninger and Lames [17] found that the long rallies with more than five strokes can be considered as the most impacting rallies in a game. One reason for these contradictory conclusions may be due to the different classifications of the indicators. In their study, the long rallies with more than five strokes is relative to the first, second, third, fourth and fifth stroke respectively. While in present research, four phases (the first and third shots, the second and fourth shots, the fifth and after shots, and the sixth and after shots) is adopted. Another reason may be because the research methods is different. They used the method of mathematical simulation, while we used multiple regression and TDC based on actual data. Other reasons for this phenomenon may need to be further studied.

#### Differences between Chinese players and players from other countries and regions

There was little difference in the importance degree between Chinese women players and women players from other countries and regions. But for Chinese men players, the shot effectiveness model was quite different from that for other players. The most important shot effectiveness of Chinese men players was SE_1_, while that of players from other countries and regions was SE_2_. This is mainly because Chinese men players have inherited traditional technical style characteristics in the first and third shots, which is named “attack after serve” and is considered the first tactical ability in China [22, 36]. Moreover, most of the men players from other countries or regions in this study are new-generation young players, such as Tomokazu Harimoto (Japan) and Hugo Calderano (Brazil), who have used offensive receiving techniques better in competition [40]; thus, SE_2_ is more important than SE_1_ for them.

In additional, the SE_3_ for both Chinese men and women players were more important than the SE_4_, However, the importance of SE_3_ and SE_4_ were almost the same for the men and women players from other countries and regions. This might also contributed to Chinese player’s technical style characteristic, they are good at taking the initiative attack on the rally I phase.

### Compensation effect between shot effectiveness

There is a compensation effect between the various shot effectiveness levels of table tennis players. The weakness of certain shot effectiveness can be compensated by other strong shot effectiveness which has been supported by other scholars [41]. Therefore, if certain shot effectiveness plays poorly, while others play well, they can still win the game. This compensation effect will vary according to the type and level of shot effectiveness, and the total evaluation score of 12 and 13 is the dividing line for success or failure in both men and women matches. These effects should be given more attention in scientific research and training.

### Practical implications of the structure of the shot effectiveness model

The importance degree of shot effectiveness and the relationships between them play a crucial role in practical training. Through a comparative analysis of men and women players, Chinese players, and players from other countries and regions, this study unveils the structure of the stroke effectiveness model for elite table tennis. In fact, the Chinese national table tennis teams have long been engaged in analysing players’ technical effectiveness as part of their preparations for international competitions [19, 25]. They have acknowledged the significance of shot effectiveness to some extent. In this study, the significance of shot effectiveness was quantified, and specific values were calculated, providing more robust scientific support for training purposes.

Furthermore, in practical training and competition planning, coaches must consider not only the importance of shot effectiveness but also the compensatory effects between them. This consideration becomes particularly pertinent when employing training methods aimed at enhancing an player’s overall performance by refining individual techniques or tactics initially [1]. Singular improvements in technique or tactic might not yield immediate positive outcomes in terms of game victories. Instead, they could potentially lead to phenomena like “practice bias” and a disconnection between training and actual matches.

Hence, obtaining a correct understanding of the structure of the shot effectiveness model for table tennis proves beneficial for coaches and players. This comprehension assists in tailoring training regimens to address specific areas, thereby fostering an elevation in the competitive level.

### Comparison with other methods

The previous studies reflecting the importance of technical and tactical aspects for elite table tennis players often involved comparisons of indicators among players of different skill levels [7, 19, 21], mathematical simulations [17], or were based on specific technical or tactical behaviors [25, 26]. These studies entailed comparing various game performances and subsequently deducing which techniques or tactics hold greater significance in achieving victory, relying on statistical findings.

In contrast to such research, this study analyzes shot effectiveness, a factor influencing the overall competition outcome, and subsequently quantifies its degree of importance. Additionally, it uniquely employs a combination of multiple regression and TDC to yield quantitative analysis results, thereby presenting the initial evidence of compensatory effects among shot effectiveness variables. These findings offer valuable insights for coaches and players in performance analysis, consequently bearing significant practical implications.

### Limitations

Firstly, this study constitutes a comprehensive analysis of table tennis matches [23, 24], devoid of specific considerations for technical and tactical variables. Consequently, the conclusions drawn are broad and relative in nature. When addressing particular issues in practical training, it becomes imperative to account for individual variations in playing styles and personality traits.

Secondly, owing to the intricacies of techniques and the adaptability of tactics within table tennis [42], the significance of shot effectiveness may undergo fluctuations under certain circumstances. Players should be flexible in their approach when facing different opponents. For instance, if an opponent exhibits exceptional SE_1_ performance, the player’s SE_2_ might not be the foremost determinant of victory. Instead, the player could potentially secure success by capitalizing on situations where they hold an advantage in their shots.

## Conclusion

We achieved the following significant results when compared to other table tennis match analysis techniques: (1) TDC could well reflect the important degree of each shot effectiveness through various ways on winning probability in table tennis matches. (2) There was little difference in the importance degree of each shot effectiveness between men and women players. The importance degrees of SE_1_, SE_2_, SE_3_, and SE_4_ for both men and women players account for approximately 25%, 35%, 22%, and 16% respectively. (3) There was little difference in the importance degree of each shot effectiveness between Chinese women players and women players from other countries and regions and regions with the same importance order of SE_2_ > SE_1_ > SE_3_ > SE_4_. However, the structure of the shot effectiveness model for men players was quite different from that for women players. The model structure for Chinese men players was SE_1_ > SE_2_ > SE_3_ > SE_4_, and for men players from other countries and regions, it was SE_2_ > SE_1_ > SE_4_ > SE_3_. (4) There is a compensation effect between shot effectiveness of table tennis players, and the magnitude of this effect will vary according to the type and level of shot effectiveness. The total evaluation score of 12 and 13 is the dividing line for success or failure in both men and women matches. A correct understanding of the structure of the shot effectiveness model for table tennis will be helpful for coaches and players to carry out targeted training, thus improving the competition level.

## Data Availability

The datasets used and/or analysed during the current study are available from the corresponding author on reasonable request.

## Abbreviations

TDC: Total decision coefficient.
SE: Shot effectiveness.
SE_1_: Shot effectiveness of the first and third shots.
SE_2_: Shot effectiveness of the second and fourth shots.
SE_3_: Shot effectiveness of the fifth and after shots in the serving round.
SE_4_: Shot effectiveness of the sixth and after shots in the receiving round.
WP: Winning probability.
VIF: Variance inflation factor.
